# How to adapt your eye service in the time of COVID-19

**Published:** 2020-09-01

**Authors:** Fatima Kyari, Elanor Watts

**Affiliations:** 1 Associate Professor: International Centre for Eye Health, London School of Hygiene & Tropical Medicine, UK and Consultant Ophthalmologist: College of Health Sciences, University of Abuja, Nigeria.; 2 Trainee Doctor and MSc Student: International Centre for Eye Health, London School of Hygiene & Tropical Medicine, London, UK.

**Eye services must adapt to prevent the transmission of SARS-CoV-2, the virus responsible for COVID-19.**

**Figure:**
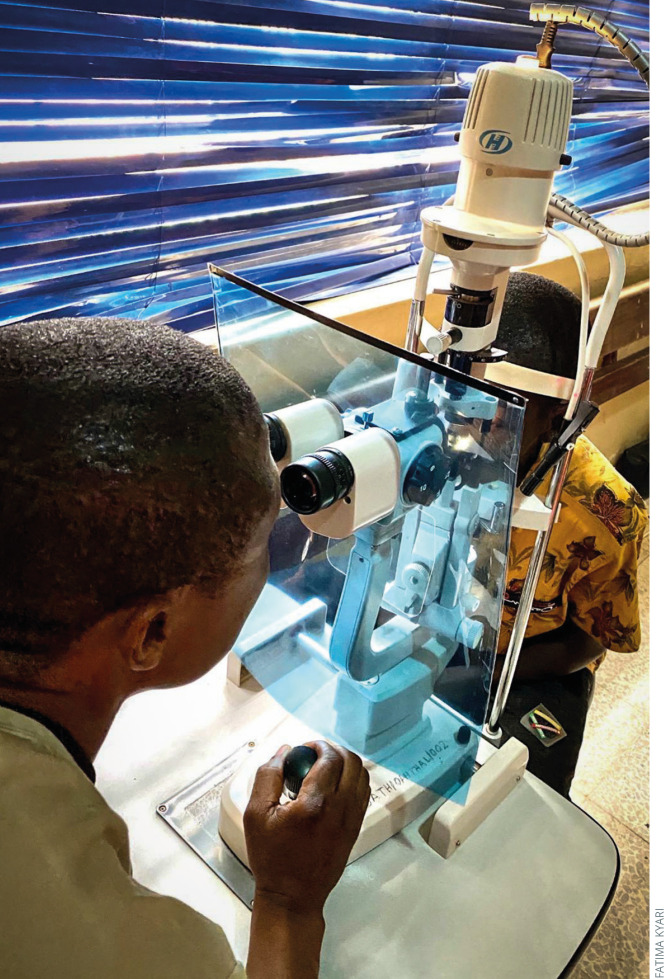
Slit lamp breath shield made from a transparent plastic folder. **NIGERIA**

Depending on national guidelines, eye services worldwide may be running a full service, postponing non-urgent eye care, or re-opening eye services once again. In this article, we discuss the practical considerations of managing COVID-19, whatever stage your country is in.

## Waiting areas and patient flow

To reduce transmission among people coming into the clinic:

1. Reduce the number of people in the clinic at any one time
2. Keep people a safe distance apart (1–2 metres)
3. Reduce opportunities for indirect transmission, e.g., when people touch objects that others have touched
4. Ensure good ventilation, which can be as simple as opening doors and windows.

**Note:** Patients with COVID-19, or who have symptoms of COVID-19, should be identified early on and seen in a separate area. Some hospitals provide a ‘red zone’ for patients with confirmed or suspected COVID-19 patients, and a ‘green zone’ for other patients.

**“Ask patients to limit the number of people going with them to an absolute minimum; preferably none.”**

### Reduce the number of people in the clinic

- Re-organise different eye clinics (e.g., glaucoma or cataract) so that as few as possible are open on the same day or at the same time. It may also help to schedule patient appointments at specific times. If this is difficult, explore innovative ways such as sending text messages ahead of time. Patient numbers will also be reduced if routine work is deferred.
- Ask patients to limit the number of people going with them to an absolute minimum; preferably none. In some settings, going with an elderly person to the hospital is a sign of respect and care, thus the advantage of fewer people needs to be explained at the time of making appointments or on arrival at the unit. Some accompanying persons will clearly still be needed for some situations, such as for children or those with visual impairment. Otherwise, consider keeping extra people out of the waiting area and the examination room.

### Keep people a safe distance apart

- Redesign seating areas to ensure adequate separation between people (Figure 1).
- In areas where people queue, create ‘boxes,’ using tape on the floor, to show where they must stand.
- Develop one-way systems for people entering and leaving the clinic so people do not have to pass one another in narrow corridors. Indicate the direction of patient flow by creating arrows on the floor. Use contrasting colours so people with visual impairment can see the arrows clearly; for example, white arrows outlined using black tape.

### Reduce opportunities for indirect transmission

- Remove from waiting rooms any objects that several people are likely to touch, such as books and magazines.
- Clean chairs and arm rests at regular intervals.
- Avoid using the same room for different purposes where possible. For example, avoid using the same room for staff members (e.g., as a seminar room) and patients at various times of the day. If this is not possible, clean rooms thoroughly between uses.
- Where possible, prop doors open so that patients do not need to touch any door handles. If this is not possible, provide hand sanitiser or hand washing facilities inside the clinic to avoid the spread of infection. Clean all door handles at regular intervals.

## Encourage good respiratory and hand hygiene

We all need to make changes in the way we live, work, and interact with people to reduce the spread of SARS-Cov-2. How we speak, cough, sneeze, rub eyes, touch our face, and touch surfaces can aid the transmission of the virus.

- Provide hand sanitisers and hand washing facilities. Patient hand washing facilities should be in a visible and easily accessible location outside the main entrance to the clinic, with hand washing facilities (or hand sanitisers) inside the clinic if needed.
  **Figure 1:**
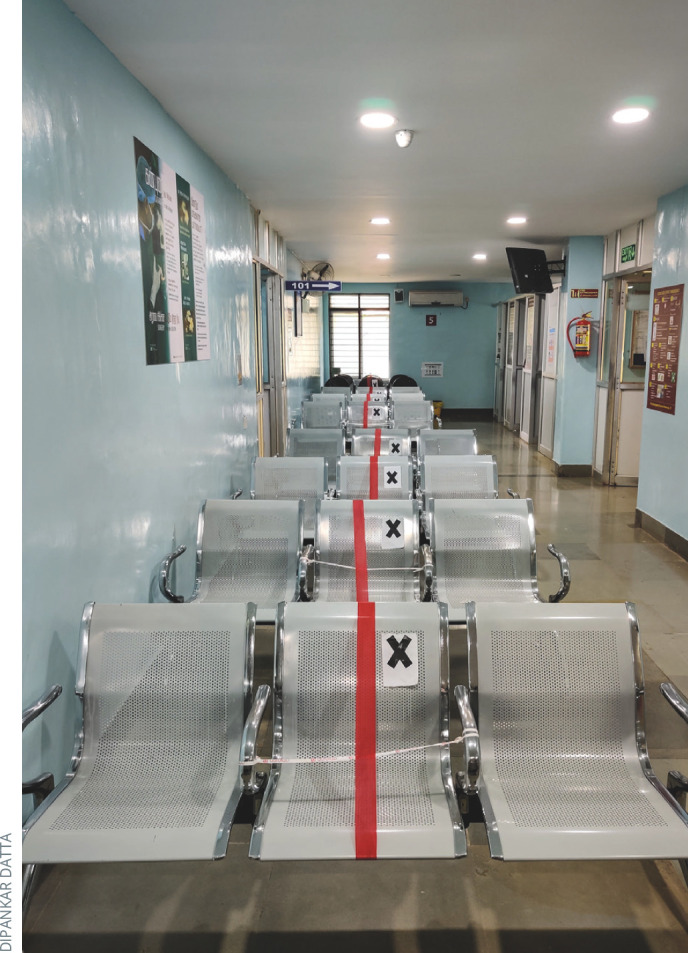
Tape across the central seats prevent patients from sitting too close to one another. The colour of the tape and the high-contrast sign (a thick, black cross on a white background) is helpful for people with vision impairment, including low vision. **INDIA**
- Consider national guidelines on the use of facemasks by patients and others coming to the clinic. Where these are recommended, advise people on the type of facemasks to use (e.g., cloth facemasks) and ensure they understand how to use and care for them. Masks are a personal item which must not be shared under any circumstances, and cloth face masks must be washed after each use.
- A more informed patient community will act more responsibly and help to lower transmission. Place posters and signs with COVID-19 information where they will be noticed. Use clear and understandable images to make the information accessible to people who are unable to read or who have low vision. Important messages include frequent handwashing and how to cough appropriately. An example of this is the **COVID-19 prevention poster** produced by the Nigerian Centre for Disease Control (Figure 2).
  **Figure 2:**
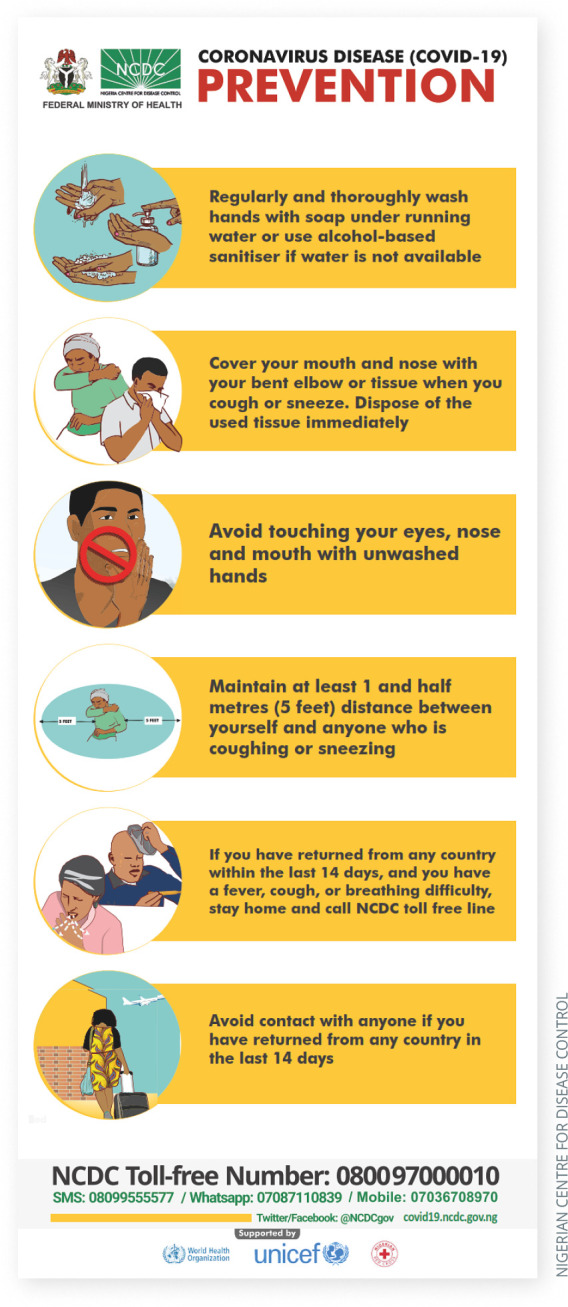
COVID-19 prevention poster produced by the Nigerian Centre for Disease Control. **NIGERIA**
- Use audio-visual messaging screens, where available, to engage patients and the people going with them.

## Seeing patients safely

Over 1,000 health workers have died from COVID-19 worldwide.^1^ Minimising the risk to health workers is important as it not only protects doctors from becoming ill, but also prevents them from passing the virus on to other patients. We recommend the following measures in addition to the proper use of personal protective equipment (PPE).

- Avoid shaking hands, or any other patient contact, as much as possible.
- Use protective equipment, e.g., large slit lamp breath shields (Figure 3). These can be bought, or in some cases received for free, from slit lamp manufacturers. Make your own using smooth, transparent plastic material (Figure 3) that is easy to clean. Use a template, such as those produced by some slit lamp manufacturers,^4^ or use paper to make your own template by tracing around the eyepiece of the slit lamp.
  **Figure 3:**
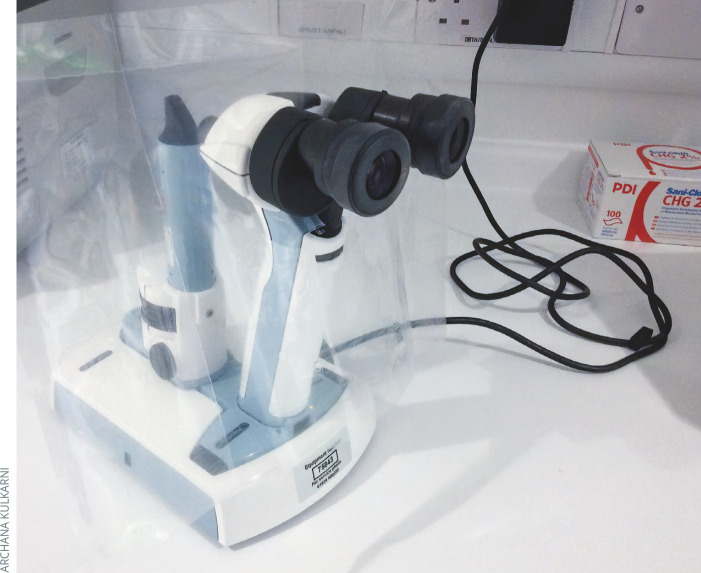
Portable slit lamp breath shield. **uk**
  **Figure 4:**
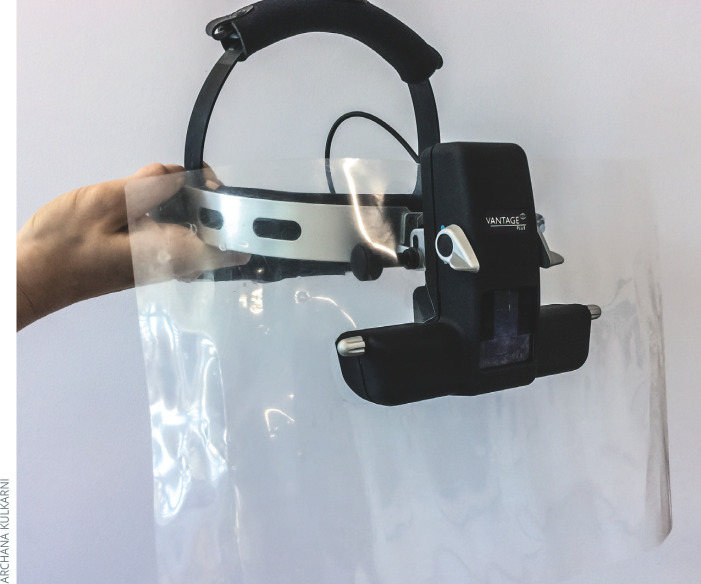
Indirect ophthalmoscope breath shield. **uk**
- Recommend that patients and clinicians avoid speaking when at the slit lamp (or keep it to a minimum), and to remain 1–2 metres apart at other times.
- Add home-made breath shields to portable slit lamps (Figure 3) and indirect ophthalmoscopes (Figure 4). Avoid using direct ophthalmoscopes.
- Clean and disinfect breath shields and ophthalmic equipment (e.g., slit lamp, tonometer, trial frames, lenses, etc.) with great care between each patient. Use disposable gloves and a solution of household bleach (1.5 tablespoons per litre of water) or alcohol solutions with at least 70% ethyl alcohol and isopropyl alcohol.
- Clean and disinfect surfaces and any areas touched by patients and health workers, such as seats, door handles, phones, keyboards, pencils and any controls/buttons on the slit lamp. Use the same solutions as for cleaning ophthalmic equipment.
- Minimise patient contact time by reviewing any notes beforehand. One option is to place the patient in one room and the clinician in another, and for them to talk via a phone or tablet computer. The clinician then enters the patient room and conducts a swift examination, with minimal speaking, before exiting and continuing the conversation as before. Another option is to hang a plastic screen between patient and health worker where it is feasible to do so.
- Keep investigations such as visual fields or OCT scans to an absolute minimum.
- Restrict the use of cash and avoid sharing personal items such as mobile phones, keyrings and pens/biros.

**Disinfectant solutions**

- 1.5 tablespoons (22.5 ml) of household bleach per litre of water
- Alcohol solution with at least 70% ethyl alcohol and isopropyl alcohol

Staff members who have any symptoms of COVID-19 should self-isolate at home, in keeping with national guidelines. Staff who are at risk of severe COVID-19 complications, such as those with medical co-morbidities or with an older age, should step down from frontline work, in keeping with national and local guidelines.

## Telemedicine consultations

Telephone consultations may have a useful role in reducing the need for face-to-face consultations. An alternative, where possible, is the use of video conferencing. This allows clinicians to see patients, which can be very helpful.

Many telemedicine software options are becoming available and have been successfully implemented in parts of India. Telemedicine will continue to play a significant role into the future,^5^ especially for those more vulnerable to severe COVID-19 complications. It also ensures that patients do not feel left behind while face-to-face services are not available.

It is important to maintain patient confidentiality and follow national guidelines regarding data protection.

## Governance

- Set up a designated team to regularly check local and national updates (e.g., the daily situation report) to stay up to date with the case definition, local predominant symptoms, and reporting/notification channels, so that suspected COVID-19 patients are managed correctly.
- Because different institutions and countries have their different health systems challenges, develop written protocols of practice setting out the changes you are making to address COVID-19. These must align with local and national guidelines and advice.
- On the use of PPE, consider the availability and allocation of resources, as well as environmental sustainability. Aim to reduce the amount of waste you produce, but without compromising infection control.
- As more clinical meetings and tutorials are undertaken as webinars, engage with regulatory medical councils and encourage them to review their rules and begin to recognise online continuous medical education for certification.

**“Staff members who have any symptoms of COVID-19 should self-isolate at home, in keeping with national guidelines.”**
