# Supplementary material for: How to adapt your eye service in the time of COVID-19
Source: Community Eye Health. 2020 Sep 1;33(109):14–7. (PMC7677804)
Supplement: Related texts

# Brief report: Future directions

**Published:** 2020

Eye care providers cannot wait indefinitely for the COVID-19 pandemic to resolve itself. As we know, COVID-19 spreads easily and rapidly, but there are non-urgent eye diseases that we need to treat. We must face this challenge, recognise and manage the scientific expectations, and redefine a new normal for the practice of ophthalmology. We recognise that eye diseases and major causes of blindness remain the same, and that treatment for those diseases remain the same. Therefore, we need to adapt our eye care delivery service to redefine the clinical encounter and develop safe delivery systems to prevent outbreaks in our place of work.

Hopefully, effective treatment options will become available soon. In the meantime, many units are now facing the challenge of when and how to restart services beyond very acute urgent and emergency work. The longer services are deferred, and eye diseases are not treated, the greater the risk of vision loss and blindness which could have been avoided.

## Tests, prevention, and treatment for SARS-CoV-2 infection

Although our understanding of the epidemiology and clinical pattern of COVID-19 continues to evolve, and clinical trials for treatment are ongoing, it is safer to assume that there is no known treatment for the disease. As of now, resources do not exist for universal testing. But, hopefully, the following options may be available in the near future:

- Antibody tests for clinicians, staff members, and patients. This will have to be an easy, reliable, affordable, and quick test which could be given to everyone and would identify individuals who are, or have been, infected so they can be isolated and/or their contacts can be traced.
- Pre-op throat swab tests. This is important in procedures considered as aerosol-generating, e.g. phacoemulsification, and procedures requiring general anaesthesia. Other options are lung CT to assess the lung parenchyma for pneumonia or fibrosis, and full blood count and inflammatory markers assay (e.g., C-reactive protein).
- Vaccination. Developments are ongoing. A vaccine for SARS-CoV-2 is still some time away.

## Medical education

In the medium to long term, eye units and facilities, supported by their professional groups/societies, need to include disaster management and simulation of case scenarios in medical education so that the service disruptions that resulted during this pandemic are kept to a minimum in the future.

## Research

There has been much speculation about conjunctivitis in COVID-19, and that the tear-film harbours SARS-CoV-2 and is transmissible during ophthalmic examination at different stages of the infection. It is important to gather evidence through population-based research on the epidemiology, transmissibility, symptomatology and clinical ocular features of COVID-19. This will contribute towards providing more appropriate patient care.

## Conclusion

Unfortunately, fragile health systems will return to the new normal in a less unified/organised manner. Where there have been no established social protection schemes, the response will be slower, and even more difficult. Nevertheless, eye services need to balance patients’ behaviour and safety with providers’ responsibilities in order to provide appropriate medical care more quickly and in a safe environment.

**Resources**

1. **Guidelines and information from International Council of Ophthalmology (ICO) member societies** **http://www.icoph.org/news/news_detail/602/ICO-Global-COVID-19-Resource-Center.html** (Last Accessed 10 May 2020).
2. Parke DW. **Ophthalmology after coronavirus disease 2019 (COVID-19): Transition back to patient care**. JAMA Ophthalmology. https://doi.org/10.1001/jamaophthalmol.2020.2004
3. **Nigerian Centre for Disease Control COVID-19 guidelines** **https://covid19.ncdc.gov.ng/guideline/**
4. **Example templates for slit lamp breath shields** **https://www.haag-streit.com/fileadmin/Haag-Streit_Diagnostics/slitlamps/Divers/Template_for_Extended_Breath_Protecting_Shield.pdf**
5. **Guidelines on the use of technology for telemedicine** **https://www.rcophth.ac.uk/wp-content/uploads/2020/05/Overview-of-digital-technology-and-telemedicine-for-covid-060520-1.docx**

## References

[1] In Memoriam: Healthcare Workers Who Have Died of COVID-19. Medscape 1 Apr 2020 (updated 13 May 2020). **www.medscape.com/viewarticle/927976**
